# The Glioblastoma Landscape: Hallmarks of Disease, Therapeutic Resistance, and Treatment Opportunities

**DOI:** 10.18103/mra.v11i6.3994

**Published:** 2023-06-26

**Authors:** Jack Boylan, Elizabeth Byers, Deborah F. Kelly

**Affiliations:** 1 Department of Biomedical Engineering, Pennsylvania State University, University Park, PA 16802, USA.; 2 Center for Structural Oncology, Pennsylvania State University, University Park, PA 16802, USA.; 3 Molecular, Cellular, and Integrative Biosciences Graduate Program, Huck Institutes of the Life Sciences, Pennsylvania State University, University Park, PA 16802, USA.

**Keywords:** Glioblastoma, Glioblastoma stem cells, heterogeneity, therapy resistance, tumor microenvironment, clinical treatments

## Abstract

Malignant brain tumors are aggressive and difficult to treat. Glioblastoma is the most common and lethal form of primary brain tumor, often found in patients with no genetic predisposition. The median life expectancy for individuals diagnosed with this condition is 6 months to 2 years and there is no known cure. New paradigms in cancer biology implicate a small subset of tumor cells in initiating and sustaining these incurable brain tumors. Here, we discuss the heterogenous nature of glioblastoma and theories behind its capacity for therapy resistance and recurrence. Within the cancer landscape, cancer stem cells are thought to be both tumor initiators and major contributors to tumor heterogeneity and therapy evasion and such cells have been identified in glioblastoma. At the cellular level, disruptions in the delicate balance between differentiation and self-renewal spur transformation and support tumor growth. While rapidly dividing cells are more sensitive to elimination by traditional treatments, glioblastoma stem cells evade these measures through slow division and reversible exit from the cell cycle. At the molecular level, glioblastoma tumor cells exploit several signaling pathways to evade conventional therapies through improved DNA repair mechanisms and a flexible state of senescence. We examine these common evasion techniques while discussing potential molecular approaches to better target these deadly tumors. Equally important, the presented information encourages the idea of augmenting conventional treatments with novel glioblastoma stem cell-directed therapies, as eliminating these harmful progenitors holds great potential to modulate tumor recurrence.

## 1. Introduction

Glioblastoma (GBM) is the most common and deadly primary brain tumor diagnosis in Europe and North America^1,2^. It is an aggressive disease characterized by rapid tumor growth, potent invasive behavior, and high therapeutic resistance. Due to its severity, the prognosis for GBM is bleak with a median survival of ~15 months and a 5-year survival rate of only 5%^3^. While there is no cure for GBM, standard treatment entails surgical resection followed by dual therapy irradiation and the adjuvant chemotherapeutic temozolomide (TMZ)^4^. Years of clinical investigation indicate this regimen can improve patient survival rates^5^. Unfortunately, this combinatorial therapy does not comprehensively clear all tumor cells^6^. As a consequence, GBM is notorious for its inevitable recurrence and readministering these interventions provides only modest benefits^7^. Several features of GBM impair therapeutic efficacy, the first of which is the blood brain barrier (BBB). Drugs that improve tumor sensitivity to TMZ have difficulty penetrating the restrictive BBB, presenting a major challenge for chemotherapeutics^8^. Further, the vasculature network is leaky in GBM tumors, contributing to insufficient drug delivery across the tumor and decreasing their efficacy^9^. Also, due to their potent invasive nature, the boundary between tumor cells and healthy cells is poorly delineated, making complete surgical resection virtually impossible^10^.

Tumor heterogeneity is another key feature of GBM. A single GBM tumor is comprised of diverse cell types harboring a variety of genetic and transcriptomic phenotypes^11,12^. High levels of heterogeneity mean differing sensitivities to therapies, impairing their universal potency^13-15^. Based on these pleiomorphic features, there is growing support for the presence of cellular populations that exhibit stem cell-like properties termed “cancer stem cells” (CSCs). Co-existing symbiotically alongside bulk tumor cells, CSCs are thought to strongly contribute to tumor development, drug resistance, and cancer recurrence^15-21^. Distinct niches or microenvironments house CSC populations and amplify signals for tumor progression and maintain stemness. In addition to intrinsic resistance mechanisms, under therapeutic stress, CSCs and bulk tumor cells can reversibly exit the cell cycle, thereby evading therapies that rely on cell division before reinitiating tumorigenesis^22^.

As the recognition and importance of CSCs continue to grow, it is imperative to understand their molecular properties, invasive behavior, and interactions with surrounding cells. This review aims to better inform clinicians and scientists entering the complex field of GBM research. Here, we summarize issues related to cellular heterogeneity, tumor microenvironment, and therapeutic resistance while exploring potential therapeutic targets aimed at the eliminating CSCs.

## 2. Heterogeneity in glioblastoma

The variety of cell phenotypes present in GBM has been observed since it’s early diagnoses. Due to GBM’s hallmark complexity, it was the first cancer to be fully sequenced by The Cancer Genome Atlas Initiative^23^ and other –omics data quickly followed^24-27^. Molecular profiling now accompanies histology in identifying and classifying GBM^28^. Sequencing showed many differently expressed genes between GBM tumors but also several common alterations now used for diagnosis. These abnormalities include amplification of the endothelial growth factor receptor (EGFR), loss of chromosome 10, amplification of chromosome 7, and mutations of the telomerase (TERT) promoter^23,27,29^. One significant mutation used for GBM classification is in the isocitrate dehydrogenase (IDH1) gene^25^. Found in ~9% of patients, this mutation impairs tumor cell metabolism and influences overall survival rate – 1.1 years for wild type IDH1 (IDH-wt) compared to 3.8 years for mutant IDH1 (IDH-mut)^30-32^. So clinically divergent are IDH-wt and IDH-mut GBMs that the 2021 WHO nomenclature redefined ‘glioblastoma-mutant IDH’ as grade 4 ‘astrocytoma - mutant IDH’^33^.

Using molecular profiling criteria, GBM tumors have been clustered into three subtypes: proneural (PN), proliferative, and mesenchymal (MES)^34^. Transcriptome and single-cell analysis of multiple GBM tumors by Patel et. al identified that transcript signatures describing these bulk tumor subtypes also exist as geographically separate regions inside a single tumor^26^. Not only can a whole tumor be classified MES or PN, but regions within that tumor obeyed the same classification and expression signature, often with more than one such region in a single tumor^26,35,36^. In addition to spatial heterogeneity, tumors can also exhibit temporal heterogeneity, with ~45 - 63% of GBM tumors changing expression signatures during evolution and in response to environmental stimuli^37-39^. One of the most relevant examples of this change is the PN to MES transition observed in response to radiation therapy^40^. The MES signature is correlated with decreased therapy sensitivity, leading to an enrichment of MES cells in recurrent GBM which contributes to its intractability^40,41^.

### 2.1. The clonal evolution theory and the cancer stem cell theory

One hypothesis to explain tumor heterogeneity is the clonal evolution theory^42,43^. In this theory, a single transformed ancestor cell divides to establish and populate the tumor, using its selective growth advantage to outcompete normal cells and pass down cancerous mutations to each new generation. Each cancer cell then possesses the same tumor-initiating mutations that transformed its ancestor while accumulating new, potentially advantageous mutations through genetic instability. Heterogeneity then is the result of this Darwinian process, with naturally selected cells creating individual regions expressing a unique, advantageous genetic and epigenetic profile. This is, however, not the only explanation for the origins of heterogeneity.

**Figure 1.**
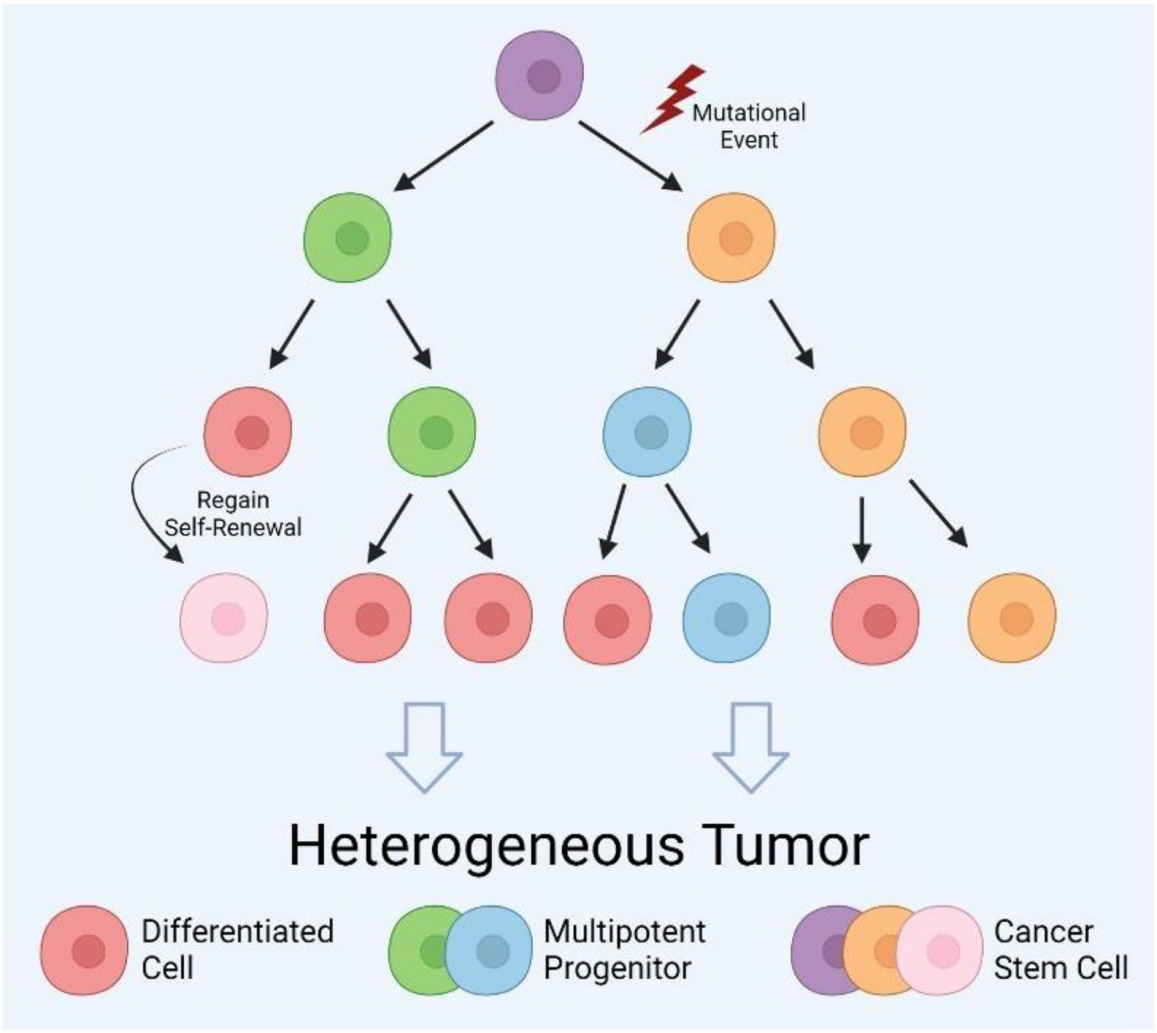
Tumor heterogeneity within glioblastoma lesions. Tumor heterogeneity can be explained by the cancer stem cell (CSC) theory. In this hierarchical model, a single transformed stem cell (purple) can self-renew to create another CSC and a rapidly cycling multipotent progenitor (blue/green). These progenitors divide to supply the tumor bulk with differentiated cells (red). Heterogeneity arises from the degrees of differentiation through the tumor. The clonal evolution theory can also be incorporated into this framework through mutational events during CSC self-renewal to create a genetically novel CSC (orange) or by dedifferentiation of a differentiated cell back into a CSC (pink). These populations of CSCs and their descendants can then compete through natural selection of growth advantages. Created with BioRender.

In addition to clonal evolution, the cancer stem cell (CSC) theory can explain tumoral heterogeneity (Figure 1). By the clonal evolution theory, each cancer cell harbors the transformative mutations to make it tumorigenic and able to replicate indefinitely^44^. In contrast, in 1997, Bonnet et al. presented evidence in acute myeloid leukemia that only a small population of tumor cells are capable of recapitulating the tumor^18^. Specifically, isolated cancer cells expressing the stem cell marker CD34 formed tumors in xenograft transplants while cells lacking this marker did not^18^. Therefore, they concluded this small malignant population were “tumor-initiating cells (TICs)” and serve as progenitors for the remaining bulk of cancer cells. This theory contradicts clonal evolution in which all cells possess tumorigenic potential^44^. In a foundational review, Reya et al. expanded this idea to explain tumor growth mechanics and heterogeneity in all cancers, coining the term ‘cancer stem cell’ and laying the foundations for the CSC theory^45^.

According to this theory, CSCs are a rare and uniquely tumorigenic population of cells with the potential to self-renew and differentiate, similar to conventional stem cells^46^. Mimicking stem cell behavior in organogenesis, CSCs are slow cycling but generate lineage restricted progenitors^47,48^ that populate the bulk of the tumor with differentiated cells. In this model, the spectrum of differentiation is the source of tumoral heterogeneity and only CSCs can recapitulate it in a new tumor. Recently, the unidirectionality of this hierarchy has been reevaluated due to evidence that differentiated cells can regain stem-like characteristics via modulation of signaling pathways^49,50^ caused by intrinsic genetic instability or environmental factors. Numerous studies in varying cancer types have shown differentiated tumor cells adopting CSC characteristics through wingless/nuclearization factor kappa B (Wnt/NF-κB) signaling modulation, growth factor release, and during the epithelial to mesenchymal transition (EMT)^51-54^. This plasticity of differentiated cells has led some to merge the clonal evolution and CSC models^55,56^, proposing that CSCs can clonally evolve. The new model suggests CSCs can undergo random genetic mutation during cell division, thereby producing heterogeneous CSC populations. Most importantly, the hierarchy is not locked in one direction and differentiated cells can also accumulate novel mutations before regaining self-renewal and making a CSC distinct from its ancestor (Figure 1). These unique CSCs and their progeny can then follow Darwinian processing described by the clonal evolution model. Despite some controversy^57-62^, CSCs, and stemness in general, is recognized as an emerging hallmark of cancer^63^.

### 2.2. Cancer stem cells in glioblastoma

**Figure 2.**
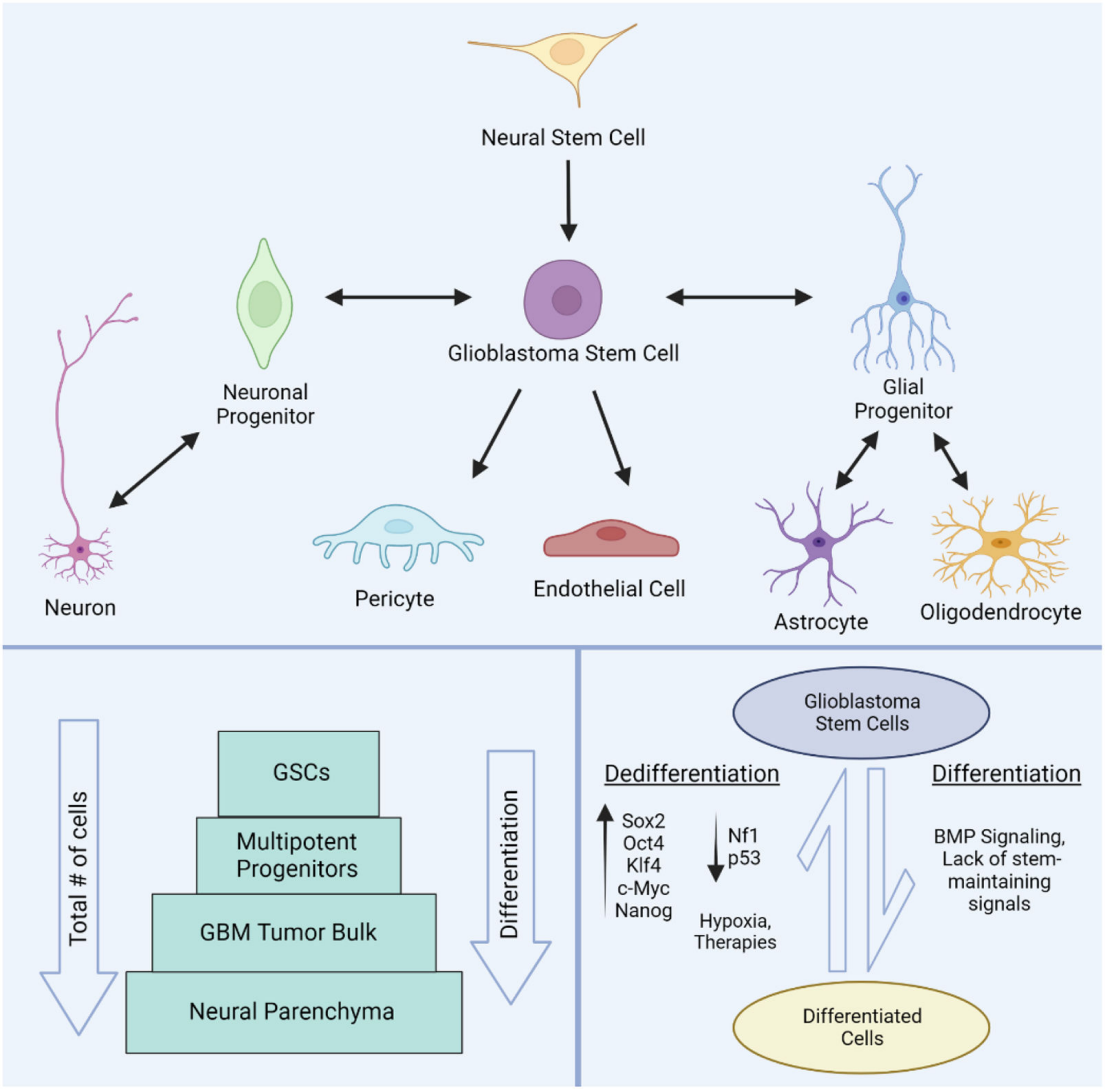
Organization and development of glioblastoma stem cells. Glioblastoma stem cells (GSCs) are multipotent and capable of differentiation into multiple cell lineages including both neuronal and glial cells. Pericytes and endothelial cells can also be differentiated from GSCs to form the expanding vasculature in GBM tumors. Glioblastoma stem cells can originate from transformed neural stem cells or from dedifferentiation of differentiated brain cells (**top panel**). Representation of the sub-populations of GSCs relative to other tumor cell constituents and differentiation status (**bottom left panel**). The conversion between GSCs and differentiated cells within tumors is regulated by a variety of signaling molecules, oncogenes, and environmental conditions (**bottom right panel**). Created with BioRender.

Cancer stem cells were identified in GBM by Singh et al. in 2003 and later termed glioblastoma stem cells (GSCs)^64^. While no single biomarker has been established to identify these cells, CD133, CD44, SSEA-1, L1CAM, Nestin and others have been used^65,66^. Satisfying the definition for CSCs, these cells possess self-renewal^67,68^, differentiate into neuronal, astroglial, oligodendroglial, and even endothelial lineages^69-71^, and initiate tumorigenesis in xenografts^64^. Originally, GSCs were believed to arise from transformed neural stem cells (NSCs) as many glioblastomas originate in the compartment housing healthy NSCs, the subventricular zone (SVZ), and they share similar gene expression profiles^64,72,73^. However, it is now understood NSC transformation is not the only origin for GSCs as differentiated brain cells have been shown to revert to a self-renewing, tumorigenic GSC state^74-76^. Astrocytes and mature neurons have been induced into GSCs by shRNA knockdown of Nf1 and p53^77^, expression of Oct4, Klf4, Sox2, c-Myc, and Nanog transcription factors^76,78-81^, and by environmental cues such as low oxygen availability (hypoxia). Alarmingly, chemotherapies and irradiation have also been shown to trigger dedifferentiation, underscoring the clinical relevance of these populations^82-84^. While GSCs do lose their stemness in response to differentiation cues like bone morphogenic protein (BMP), cells differentiated from GSCs display incomplete terminal differentiation and more easily reenter the cell cycle or dedifferentiate and form new GSCs, further amplifying heterogeneity^85,86^ (Figure 2).

Glioblastoma stem cells are crucial in tumor expansion, maintenance, and survival. Through an upregulation of genes involved in migration and extracellular matrix degradation, GSCs are more invasive than their differentiated peers^87^. In addition to expanding tumor boundaries, deep GSC infiltration makes complete surgical resection difficult^10^ and as few as 50 GSCs are capable of tumor recurrence^88^. Equally troublingly, GSCs exhibit strong chemo and radioresistance^89,90^ with high GSC numbers correlating to decreased therapy response and negative outcomes^91,92^. Recurrent GBMs are therefore enriched in GSCs^90,93^. Their DNA repair pathways are upregulated and along with their slow cell cycling, GSCs have ample time to repair therapy-induced DNA lesions, negating their cytotoxic effect^90,94^. Overall, GSCs are implicated in filling the tumor with bulk differentiated cells^47,95^ and driving tumor growth and survival. To facilitate this progression, these cells both influence and are influenced by their environment.

## 3. The tumor microenvironment and supporting niches

**Figure 3.**
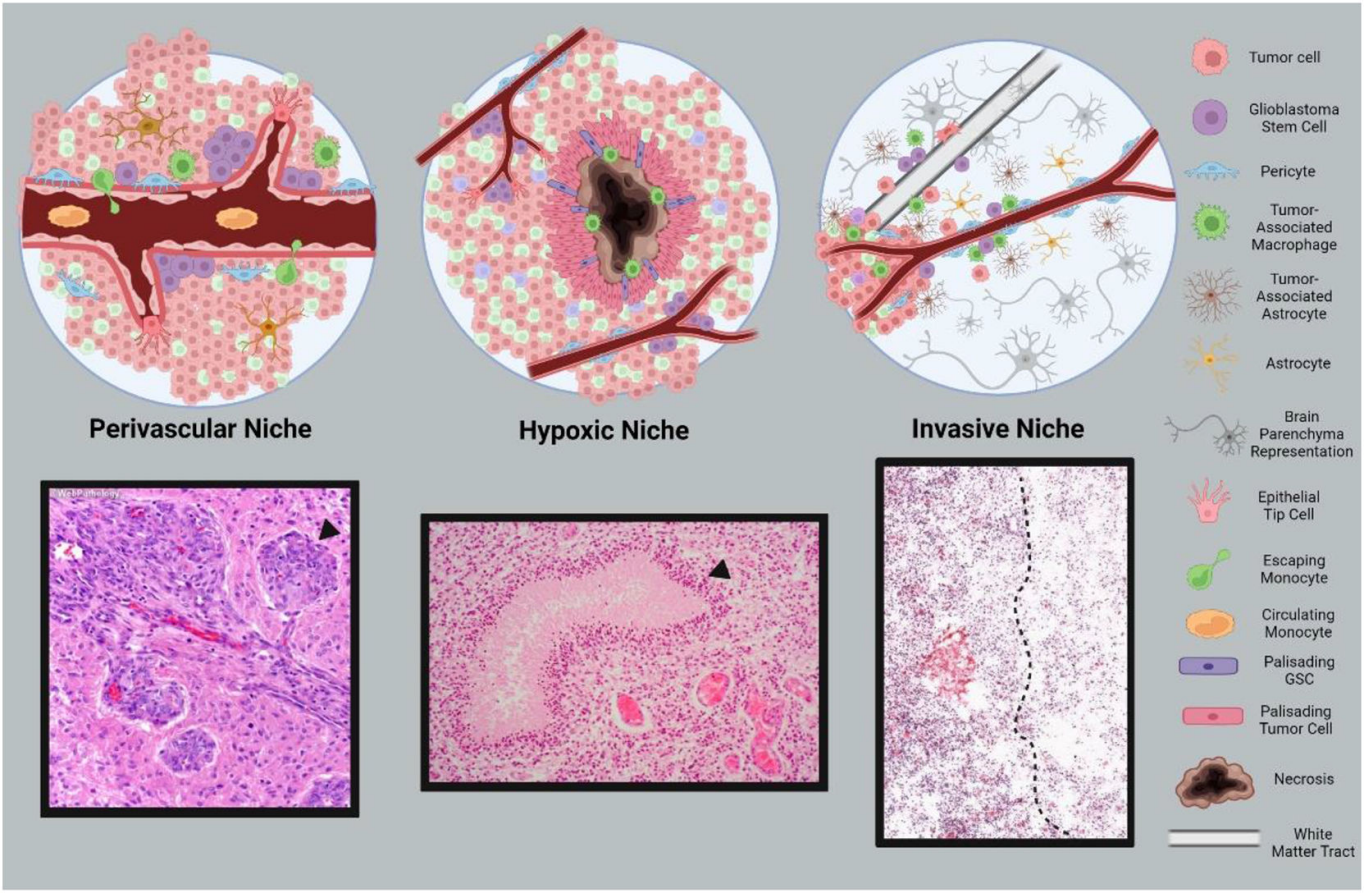
Niches that define the glioblastoma tumor microenvironment. Defined regions within the tumor microenvironment are contained within specific niches. Glioblastoma tumors contain three major niches including the perivascular niche (**left**), the hypoxic niche (**center**), and the invasive niche (**right**). A variety of cell types and populations comprising each niche are depicted in the top panels. Corresponding histological features of niche components are displayed below each schematic. Denoted are: a glomeruloid microvascular proliferation (**bottom left**), pseudopalisading cells circumscribing necrosis (**bottom center**), leading tumor edge (**bottom right**). Histology images adapted from online resources^98-99^. Created with BioRender.

Tumor cells, non-tumor cells, and other biomolecules are constantly interacting within the bulk tumor and surrounding space defined by the tumor microenvironment (TME). Highly specialized zones within TMEs are defined as specific niches. Many cancers, including GBM, contain three major CSC niches: the perivascular region, the hypoxic zone, and the invasive niche^96,97^ (Figure 3).

### 3.1. The perivascular niche

As a tumor grows, rapid cellular expansion eventually outpaces existing blood vessels’ supply capacity. New blood vessels are created through the process of angiogenesis to deliver the oxygen and other nutrients required to sustain the existing cell mass and fuel further development. Cells lacking adequate blood supply enter hypoxia, a state of oxygen deprivation, which triggers signaling responses that spur angiogenesis^100,101^. With a lack of available oxygen, the hypoxia inducible factor (HIF1/2) transcription factors are stabilized and upregulate vascular endothelial growth factor (VEGF), fibroblast growth factor (FGF), and platelet-derived growth factor (PDGF), all important angiogenesis agents^102^. Release of VEGF triggers matrix metalloproteinases (MMP) to remove the reinforcing pericytes coating blood vessels and induce remodeling^103^. Signaling molecules from the Notch pathway then cause leading endothelial cells to morph into tendrilled endothelial tip cells which extend in the direction of the VEGF signal, recruiting new endothelial cells and pericytes to construct blood vessels^104^.

Excessive VEGF-triggered angiogenesis can cause chronic vascular hyperplasia (CVH), a condition where excessive endothelial cell recruitment generates crowded, circular bundles of blood vessels termed glomeruloid microvascular proliferations (GMPs). This aberrant vasculature is a common histopathological feature of GBM often used for diagnosis^,105,106^. Importantly, because of its rapid growth, the new vascular structures are poorly assembled and prone to leakage and collapse^107,108^.

Small numbers of Nestin^+^/CD133^+^ GSCs are positioned in perivascular niche adjacent to existing vasculature and interact with endothelial cells to exacerbate angiogenesis while maintaining their stemness^109^. GSCs produce elevated levels of VEGF which accelerates and worsens angiogenesis^110^. Correspondingly, tumors generated in nude mice using CD133^+^ cells show increased vascular growth and a higher number of branching points than CD133^−^ tumors^110^. The perivascular niche may also be a refuge for GSCs during therapies as DNA repair capacity, and therefore resistance, is elevated in this region^111^.

### 3.2. The hypoxic niche

While hypoxic stress and necrosis may be expected to inhibit tumor growth, the opposite reaction occurs in GBM which develops a hypoxic niche with pseudopalisading tumor cells surrounding and escaping from a central site of hypoxia-induced necrosis^112^. The extent of necrosis is positively associated with a more severe prognosis^113^. Hypoxic conditions greatly support GSC stemness through transcription upregulation of stem-promoting genes such as *Sox2, Oct4, NANOG, Klf4 and c-Myc* while decreasing differentiation signals like BMPs^114-116^. Mesenchymal GSCs are especially sensitive to HIF2α signaling through CD44 which may explain the severity of the MES signature^117^. Hypoxia also pushes GSCs to be more resistant to therapeutics^118-121^. Following cessation of chemotherapeutics, the surviving GSCs repopulate the tumor and can adopt a therapy-resistant state to evade immune attack and preserve GSC stemness^93^.

In addition to contributing to hypoxic conditions, porous blood vessels produced during cancer-related angiogenesis enable circulating immune cells, predominantly bone marrow-derived monocytes, to enter the brain^112,122^. After squeezing through leaky vessels, the tumor environment converts these monocytes into tumor-associated macrophages (TAMs)^123,124^ which promote oncogenesis, tumor proliferation, and contribute to tumor survival^125-128^. In addition, necrotic cell death causes proinflammatory signals such as interleukin 6 (IL-6), VEGF, and stromal cell derived factor 1 (SDF-1) to be released in the tumor^129,130^. These factors promote monocyte polarization into M2 macrophages, imparting immunosuppressive effects and disabling the monocyte’s ability to clear the necrotic debris^127,131^. Hypoxia itself, through HIF1α activity, improves the immune suppression activity of TAMs and accelerates their polarization^132^. Release of interleukin 1 (IL-1) by TAMs disrupts the BBB and allows for more immune cell invasion, fueling a vicious cycle of immune cell recruitment, immunosuppression, and tumor development^133^.

### 3.3. The invasive niche

At the edge of the growing tumor is the invasive niche, where the tumor contacts normal brain tissue containing astrocytes, neurons, and extracellular matrix (ECM), among other brain features. Astrocytes comprise 50% of brain tissue^134,135^, and when the CNS is injured, astrocytes convert from their normal quiescent state into a reactive state through astrogliosis^136^. Reactive astrocytes upregulate the production of growth factors, including VEGF, cytokines (especially IL-6), and MMPs, which help the brain recover from injury by activating numerous pathways including phosphoinositide-3 kinase/protein kinase B (PI3K/AKT), Sonic hedgehog (Shh), p53, and NF-κB^137,138^. Cell sorting has shown converted astrocyte populations surrounding and inside GBM regions with a unique astrogliosis-associated transcriptome termed tumor-associated astrocytes (TAA)^135,139,140^. Within TAAs, the same signaling pathway proteins normally responsible for repair post-injury promote GBM invasion and/or proliferation^134,141^. In-vitro studies have shown an increase in proliferation and invasion when cancer cells are cultured in astrocyte-conditioned media^142-144^. TAAs also show high levels of connexin-43 (CX43) and Janus kinase/signal transducer and activator of transcription (JAK/STAT) signaling to enhance cell survival by inhibiting apoptosis and promoting immunosuppression^145^.

The resident GSC population is an active participant in the invasive niche with Nestin^+^ cells often found on the leading edge^146,147^. White matter tracts, which are utilized for migration, express Jagged1, activating Notch signaling and upregulating the transcription factor Sox2^148^ which creates a stemness-favoring environment that encourages GSC migration along the tracks. Many of the products secreted from TAAs also act to enforce cell stemness. For example, Shh signaling activates the Gli transcription factor which promotes stemness and self-renewal^149^. Upregulation of IL-6 and STAT3 signaling by TAAs is critical for stem maintenance, while STAT3 knockdown eliminates GSC multipotency and proliferation^150,151^. Macroscopically, the larger the area GBM invades, the more GSCs escape resection. As GBM can be revived by small colonies of GSCs even after treatment, the expansion of GSCs outside the tumor bulk will allow relapse.

GBM niches are highly complex and have been the focus of much research. Significantly, niches are dynamic in their temporal and spatial properties. As the invasive edge expands outward, new perivascular niches are assembled in its wake and can eventually expand the necrotic region. As niches change during GBM progression, GSCs accumulate and fluctuate, especially when exposed to therapies. The robust nature of GSCs within the different niches are thought to improve tumor cell survival and assist with therapeutic resistance.

## 4. Therapy evasion and resistance

### 4.1. Ionizing radiation resistance

As a complement to surgical resection for GBM treatment, radiation and chemotherapeutics are employed to preferentially inflict DNA damage to cancer cells. Cancer cells are vulnerable to DNA damage due to their inherent genomic instability, DNA stress from unrestrained proliferation, and mutated damage response proteins^152^. Ionizing radiation (IR) therapy bombards the tumor area with high energy particles that directly damage the DNA backbone^153^. Additionally, the high energy particles instantaneously create reactive oxygen species (ROS),such as hydroxyl radicals, inside cells and trigger mitochondria to increase their own ROS production^154^. These radicals oxidize DNA bases to form double stranded breaks (DSBs), a form of damage monitored by the ataxia telangiectasia mutated (ATM) kinase. Upon encountering DSBs, ATM kinase is phosphorylated and helps facilitate cell cycle arrest at the G1/S phase, utilizing cell cycle checkpoint 2 (CHK2) and p53^155^. Additionally, ATM acts as a scaffold to recruit repair elements to the DSB with an increase in phosphorylated ATM associated with better DNA repair^155^. A parallel system using ATM and Rad3-related protein (ATR) and CHK1 acts on single strand breaks (SSBs) in DNA and regulates entrance from G2 into M phase. Both axes pause the cell cycle for DNA damage repair and to prevent apoptosis. While elevated DNA damage fuels genomic instability, once the cancer phenotype is achieved, tumor cells rely more strongly on repair mechanisms for survival^156,157^. The diverse genetic makeup of GBM means that radioresistance in cells will also be variable in nature.

It’s no surprise that GSCs are a main culprit of radioresistance as they possess a greater ability to survive than their more differentiated counterparts. Following IR dosing, GSCs, identified as CD133^+^ cells, exhibited less apoptosis, higher ATM kinase activity, and an equal tumor-forming capacity as non-irradiated CD133^+^ cells^90,158^. Other upregulated repair genes in GSCs includes ATR, CHK1, and Poly ADP ribose polymerase (PARP1)^158,159^. As a result, it is common following IR therapy to see an enrichment in CD133^+^ populations in the tumor environment^160^. Since these cells maintain their differentiation capabilities, they can proceed to repopulate the tumor^91,161^. This new population often retains radioresistance from their surviving GSC ancestors. Finally, autophagy is suspected to play a role in radioresistance. As a defense mechanism, autophagy is triggered by cell stress such as hypoxia or elevated ROS and markers for autophagy such as LP3 and ATG5 and ATG12 increase in CD133^+^ cells following IR therapy^162^.

### 4.2. Chemotherapeutic resistance

In addition to IR therapy, GBM cells are often resistant to chemotherapies, including temozolomide (TMZ), the foundational adjuvant chemotherapeutic prescribed for GBM. Capable of penetrating the BBB, TMZ is an alkylating agent that interacts with DNA to preferentially methylate the N3 position of adenine and the N7 and O6 positions of guanine^163^. Creation of O6-methylguanine, although only 5-10% of the modifications made by TMZ, is the most cytotoxic of TMZ’s effects^163^. O6-methylguanine leads to a nucleotide mismatch during DNA replication where thymine is incorrectly inserted instead of cytosine. During repeated cycles of mismatch repair, DNA breaks are generated, causing cell cycle arrest at the G2/M transition and eventual apoptosis^164^.

The most well characterized and arguably most important mechanism of alkylating agent resistance occurs through O6-methylguanine-DNA methyltransferase (MGMT), a mismatch repair protein that repairs the damage caused by TMZ. Unfortunately, MGMT is one of the most common differentially expressed proteins in GBM tumors, meaning that many patients possess some immunity against TMZ treatment. In fact, because of the prevalence of MGMT upregulation, TMZ effect is reduced in ~50% of patients^163^ and GSCs possess greater MGMT and DDR mechanisms, making them more tolerant to TMZ treatment^94,165^. Tumors with diminished MGMT show better upfront reaction to TMZ and exhibit longer progression-free survival. Decreased levels of MGMT have been shown to be related to methylation status of the MGMT promoter, with a methylated promoter transcribing less MGMT and an unmethylated promoter more^166^. This epigenetic marker can be easily identified using methylation-specific PCR and is now considered a useful and accurate predictor of TMZ efficacy^167,168^.

### 4.3. Resistance via senescence

**Figure 4.**
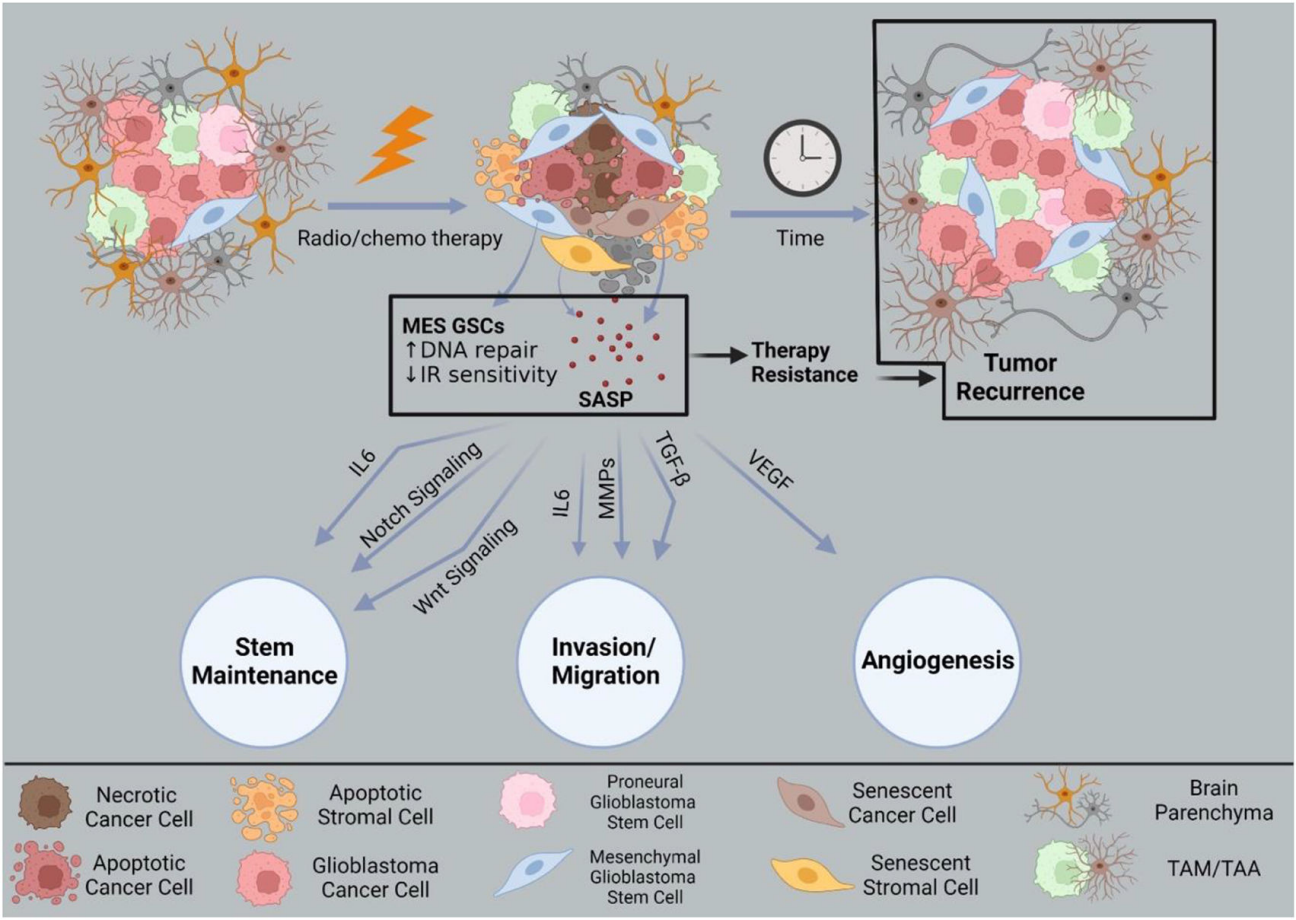
Therapy evasion of glioblastoma cells through senescence. Glioblastoma cells (top left) that resist radio/chemotherapy are enriched for therapy-resistant mesenchymal (MES) signatures. Some assume therapy induced senescence, bypassing normal apoptosis processes. Senescent tumor cells (top middle) over time can recur to give rise to new masses (top right). During senescence, cells secrete cytokines, chemokines, matrix metalloproteinases, and other growth factors known as the senescence-associated secretory phenotype (SASP). These collective signals support stem maintenance by activating the Wnt pathway. Factors from the SASP can also contribute to invasion, migration, and angiogenesis supporting tumor recurrence. Created with BioRender.

Some cancer cells do not undergo apoptosis in response to therapy-induced DNA damage and instead adopt therapy induced senescence (TIS)^169^. Senescent cells exit the cell cycle at the G2/M phase and upregulate DNA repair and apoptosis survival signals^170^. While conventional understanding suggests senescence is permanent, some studies indicate senescent tumor cells can reenter the cell cycle to become more aggressive^171^. During senescence, tumor cells secrete cytokines, chemokines, matrix metalloproteinases, and growth factors collectively known as the senescence-associated secretory phenotype (SASP)^172^ (Figure 4). In addition to fueling proliferation and invasion, the SASP also assists with GSC maintenance by activating Wnt signaling to maintain self-renewal^22,173^. The SASP can reprogram non-senescent cells to possess stem cell like qualities by releasing factors that modulate the transcription factors Oct4, Sox2, Klf4, and c-Myc, as seen in other cancers^22,173-175^.

## 5. Therapeutic avenues and clinical trials

### 5.1. Receptor tyrosine kinase signaling as a therapeutic target

Targeting the molecular pathways implicated in GBM pathology is a promising avenue for therapy development (**summarized in** Table 1). Proteins of interest include receptor tyrosine kinases, stem maintenance factors, and angiogenic markers. Crosstalk among these pathways is also an important consideration since the combination and balance/imbalance of signaling effects drives cell growth, proliferation, stemness, immune evasion, and invasion. Among the most appealing targets are the receptor tyrosine kinases (RTKs) such as EGFR and its downstream effector PI3K/AKT. Malignant amplification of these proteins is common in GBM cells with EGFR mutations occurring in 57.4% of GBM^27^. Enhanced EGFR signaling in GBM supports proliferation, angiogenesis, invasion, and survival. Small molecule RTK inhibitors that target receptors like EGFR have shown promise in other cancer types along with GBM^176,177^. Erlotinib is one such FDA approved RTK inhibitor for non-small cell lung cancer where it improved progression-free survival^178^. In combination with TMZ and IR therapy, Erlotinib showed some improvement in overall survival for newly diagnosed GBM patients, although its use as a monotherapy falls short^179,180^. Another promising EGFR inhibitor, Gefitinib, showed a decrease in EGFR phosphorylation, but this reduction did not translate to a reduction in tumor severity^181^. A combination of both Erlotinib and Gefitinib, however, was shown to be ineffective in GBM tissue samples^182^. Similarly, second-generation EGFR inhibitors Dacomitinib and Afatinib failed to show meaningful improvement as single-agent treatments^183,184^. A third-generation EGFR inhibitor, AZD9291 (osimertinib), was approved by the FDA for non-small cell lung cancer and is being evaluated for GBM with positive preclinical results^185-187^.

In addition to small molecule inhibitors, monoclonal antibodies against EGFR have also been explored, namely Cetuximab. Although most patients experienced minimal benefit, a small population showed some improvement, though the molecular underpinnings of the improvements are unclear^188,189^. Combined with standard therapy, the monoclonal antibody Nimotuzumab increased survival and tolerability in Phase II trials of MGMT-methylated GBM^190,191^. Due to their size, antibody-based therapies have difficulty breaching the BBB with only 0.1-0.2% of circulating antibodies showing BBB penetration^192^. New engineering approaches to overcome this limitation are themselves a novel field for therapeutic development^193-195^.

The PI3K/AKT/mTOR pathway is another popular frontier for drug design. Buparlisib (BKM-120) and PX-866 are two pan-PI3K inhibitors that affect multiple PI3K isoforms. Buparlisib induces apoptosis through mitotic disfunction and reduced tumor growth while increasing survival in mice with xenografted GBM tumors^196,197^. In recent clinical trials, the drug was deemed to be less effective^198,199^. Buparlisib is still being explored as a combination therapy alongside the VEGF inhibitor, Bevacuzimab^200^ and the PARP inhibitor Rucaparib^201^. Similarly, PX-866 demonstrated powerful effects in GBM cells but came up short in clinical trials with a similarly vexing absence of explanation^202,203^. Another avenue being explored is targeting individual PI3K isoforms, as it has been suggested that each isoform plays stronger roles in certain cancer characteristics^204^. Individually targeting one isoform may be specific enough to avoid redundancies while maintaining therapeutic efficacy^205,206^. Downstream from PI3K targets, new mTOR inhibitors are being developed for clinical trials^207^. They may have value in a combination therapy if monotherapies are unsuccessful. The well-tolerated drug, Perifosine, targets AKT and is under investigation in combination with PI3K and mTOR inhibitors^208^. Drugs like Temsirolimus and Everolimus that target mTOR suffer from the same theme plaguing most inhibitors of this pathway: monotherapies fail due to insufficient intertumoral drug levels or compensatory signaling^209,210^.

### 5.3. Notch, Wnt and Sonic hedgehog targets

Other potential targets in GSC maintenance include Notch, Wnt, and Shh. Notch signaling is part of neural stem cell compartmentalization, preventing apoptosis and ensuring a stock of stem cells during brain development^211^. The Notch pathway also has a protective role in GSC self-renewal^211^. Gamma-secretase inhibitors (GSIs) are used to inhibit activation of the Notch signaling pathway and early reports indicate their effectiveness at depleting GSCs or sensitizing them to radiotherapy^212-214^. Clinical trials using the GSI MK-0752 for refractory CNS malignancies (including GBM) showed good tolerance and favorable Notch inhibition^215-217^. Another Notch inhibitor, RO4929097, successfully induced differentiation and decreased self-renewal in GSCs in orthotropic mouse models^218^. In a Phase 0/1 combinatorial trial of RO4929097 with TMZ and radiotherapy, Notch signaling was diminished and explanted tumors showed decreased CD133^+^ markers. Gene expression analysis of recurrent tumors in the treatment group revealed adaptive Notch downregulation but the upregulation of mesenchymal genes and angiogenic factors as compensatory mechanisms^219^. The modulation of Notch with MK-0752 affected PI3K/AKT while RO4929097 altered VEGF levels^220,221^, a good indication that targeting several pathways simultaneously may be a productive path forward. Notably, GSIs have the potential to impair Notch regulation in healthy cells as well as in tumor cells^222,223^. As such, new generations of GSIs are being developed and tested in GBM cell lines^224^ with the hope of reducing impacts on healthy tissue.

Another upregulated pathway in GBM is the Wnt network, having roles in stem maintenance, therapy evasion, proliferation, and invasion^225-227^. Due to crosstalk between Wnt and cyclooxygenase-2 (COX-2) pathways, non-steroidal anti-inflammatory drugs (NSAIDs) that inhibit COX-2 also suppress Wnt signaling^228,229^. Small molecule inhibition with the NSAID celecoxib sensitized GBM tumors to TMZ by regulating the expression of MGMT^225^. Celecoxib was also effective at sensitizing tumor-derived GSCs to radiotherapy and increased the mean survival rates of mice with xenografted tumors^230^. In one clinical trial of 28 patients with recurrent GBM, treating with celecoxib and continuous low dose TMZ showed improvements compared to alternating TMZ dosage, with median survival time rising from 11.1 months to 16.8 months^231^. An additional clinical trial incorporating celecoxib in a multi-drug cocktail accompanied by TMZ showed anti-neoplastic effect, though the effect of celecoxib in this result is unreported^232^. Moreover, Wnt inhibitors continue to be explored in other solid tumors^233^.

Sonic hedgehog signaling mediates stem cell differentiation, and its overexpression is implicated in sustaining GSCs, providing therapy resistance, and promoting GBM invasion^234-236^. The transcriptional effector Gli1 in Shh cross-talks with the PI3K/AKT and VEGF pathways^237^. Clement et al. sensitized GSCs to TMZ through blockade of Shh using cyclopamine in GBM cell lines^149^. A dual approach with a Shh pathway inhibitor, NVP-LDE-225, and a PI3K/mTOR inhibitor, NVP-BEZ-235, diminished proliferation, growth, and stemness of isolated GSCs^238^, demonstrating the benefits of Shh inhibition. Furthermore, another Shh inhibitor, Vismodegib, is among five drugs under investigation in the NCT Master Match, an ongoing clinical trial for newly diagnosed GBM patients for whom TMZ is excluded^239^. Vismodegib has also showed promising anti-cancer activity in medulloblastoma clinical trials^240^.

### 5.4. Targeting angiogenesis and DNA repair

Angiogenesis targets such as VEGF are highly appealing to minimize nutrient delivery to GBM tumors. The humanized monoclonal antibody, Bevacuzimab, binds the VEGF ligand to prevent it from interacting with cell-surface receptors^241^. In a Phase II clinical trial, Bevacuzimab elicited a decrease in tumor size, lessened cerebral edema, and offered longer benefits to patients with recurrent GBM^242^. In light of this success, the FDA granted Bevacuzimab accelerated approval as both a monotherapy and in combination with the cytotoxic agent Irinotecan^243^. Full authorization was granted in 2017. Some GBM subtypes may decrease Bevacizumab efficacy in GBM as GSCs persist to enable therapy resistance and invasion^244-246^. Anti-angiogenesis treatments also enhance delivery of other therapeutics by repairing the leaky vessels needed to transport drugs to the tumor^247^. For example, the addition of an anti-VEGF agent was shown to improve delivery of immunotherapeutic CAR-T cells to tumors in mice^248^. This is especially valuable to reach the outer, invasive edges of the tumor^249^. Importantly, appropriate dosing is needed to balance angiogenesis prevention as over-inhibition will create necrosis through lack of nutrient supply and trigger increased tumor invasion and worse prognosis^250,251^. Despite potential risks, normalization of the vasculature through anti-angiogenic means continues to be a promising avenue to improve chemotherapy^252^.

Finally, DNA repair mechanisms are being explored as an approach for most cancer treatments including GBM. By disabling the machinery needed to overcome DNA damage, GBM tumors are sensitized to the cytotoxic effects of alkylating agents such as TMZ and the nitrosureas, lomustine and carmustine. PARP inhibitors have also become a popular sensitizing agent under considerable investigation. These agents disable the base excision repair mechanism that GSCs rely on for single strand break repair and improve radiotherapy effectiveness^253,254^. Efficacy in xenografts depends on the ability of PARP inhibitors to penetrate the BBB^255,256^. In clinical trials, the PARP inhibitor ABT-888 failed to overcome TMZ resistant in GBM, but modifications to PARP treatment remains an active area of research^257,258^. Likewise, MGMT is a main culprit chemoresistance and is a popular DNA repair target being explored for combinatorial treatments. Drug-induced knockdown of MGMT may improve outcomes for patients with unmethylated MGMT. In addition, proteosome inhibitors, such as Bortezomib, can deplete MGMT levels leading to prolonged survival in mouse models with unmethylated MGMT promoters^259,260^. An initial clinical trial showed adequate tolerance and efficacy in newly diagnosed GBM, though MGMT-methylated patients show a greater effect^261^.

## 6. Conclusions

Many challenges remain as we seek to reduce the burden of GBM in newly diagnosed patients and those experiencing recurrence. Historically recognized hurdles such as heterogeneity and therapy resistance have been reexamined with the discovery of cancer stem cells. The development of the cancer stem cell theory offers putative explanation for tumor initiation and the origin and prevalence of the highly damaging heterogeneity observed in GBM. Through this heterogeneity, therapies are less universally effective. Inclusion of GSCs into the GBM model also offers further explanations for GBM’s radiation and chemotherapeutic evasion as GSCs possess improved DNA repair mechanisms, a slower cell cycle, and reversible senescence phenotype, all of which allow them to overcome therapies' cytotoxic effects.

As modern paradigms in cancer biology implicate GSCs in sustaining incurable GBMs, research aimed at eliminating this sub-population is imperative. New therapeutic approaches targeting GSCs alongside bulk tumor cells may enhance treatment outcomes. Many clinical trials have been performed and more are currently underway that target pathways critical to GSC function such as RTKs, angiogenesis, DNA repair and the stem-maintenance pathways Notch, Wnt, and Shh. Compared to other cancer types, brain tumors have to navigate the restrictive BBB which limits the delivery of novel drugs, but new drug delivery may facilitate penetrance across the BBB. As a result, therapies that prove effective in eliminating GSCs can likely be easily applied to CSCs in other cancer types.

Finally, new pharmacological discovery tools are needed to close the gap between cellular success stories and those of clinical trials. Better structure-activity-relationship predictive tools are imperative to elevate rational drug design based on native protein structures. The combined use of automated drug screening tools and cryo-Electron Microscopy (EM) structure determination creates a new era in therapeutic development. As many protein structures are now amenable to three-dimensional analysis at high-resolution, the next wave of drug design may include pinpointing protein binding sites to improve inhibitors of signaling pathways or DNA repair. Overall, improved outcomes for GBM patients are expected through the use of new platforms to target GSC-specific properties at the nanoscale. Future research efforts aimed at eliminating GSC self-renewal is a necessary consideration for scientists and clinicians working in the GBM field.

## Figures and Tables

**Table 1.** Summary of molecular drug therapies in glioblastoma treatment.

Pathway	Drug therapy	GBM status and treatment	Efficacy	Ref	Clinical Trial ID
EGFR (drug)	Erlotinib	Newly diagnosed and recurrent	Minimally effective as a mono therapy	Raizer^180^	NCT00045110
	Erlotinib + IR + TMZ	Newly diagnosed	Improved PFS and OS	Prados^179^	NCT00187486
	Gefitinib	Recurrent	Ineffective in reducing EGFR pathway	Hegi^181^	NCT00250887
	Dacomitinib	Recurrent	Ineffective as a monotherapy	Sepúlveda-Sánchez^183^	NCT01520870
	Afatinib	Recurrent	Moderate improvement for EGFRvIII^+^ patients	Reardon^184^	NCT00727506
	AZD9291 (osimertinib)	Advanced NSCLC with EGFR inhibitor resistance	Improved PFS, highly active	Jänne^185^	NCT01802632
EGFR (antibody)	Cetuximab	Recurrent	Ineffective as a monotherapy, minority saw modest OS improvement	Belda-Iniesta, Neyns^188,189^	[N/A]
	Nimotuzumab + IR + TMZ	Newly diagnosed	Improved PFS and OS	She, Du^190,191^	[N/A]
PI3K	Buparlisib	Recurrent	Minimally effective as a monotherapy	Wen^199^	NCT01339052
	Buparlisib + Bevacuzimab	Recurrent	No additional effect compared to Bevacuzimab alone	Hainsworth^200^	NCT01349660
	PX-866	First recurrence	No reduction in PI3K signaling	Pitz^203^	NCT01259869
AKT	Perifosine	Recurrent	Ineffective as a monotherapy	Kaley^208^	NCT00590954
	Perifosine + Temsirolimus	Recurrent	Ongoing	Lassman^182^	NCT02238496
mTOR	RMC-5552	Recurrent	Ongoing	Burnett^207^	NCT05557292
	Temsirolimus + Erlotinib	Recurrent	High toxicity, no reduction in AKT pathway	Wen^209^	NCT00112736
	Everolimus + IR + TMZ	Newly diagnosed	Moderate toxicity, no appreciable survival benefit	Ma^210^	NCT00553150
Notch	MK-0752	Advanced solid tumors	Variable induction of stable disease	Krop^217^	[N/A]
	RO4929097 + IR + TMZ	Newly diagnosed	Tolerable toxicity, CD133^+^ cells diminished	Xu^219^	NCT01119599
	RO4929097	Recurrent	Ineffective as a monotherapy	Peereboom^262^	NCT01122901
Wnt/COX2	Celecoxib + low dose TMZ	Recurrent	Improved benefit compared to IR + TMZ alone	Stockhammer^231^	[N/A]
Shh	Glasdegib + IRT + TMZ	Newly diagnosed	Promising preliminary effects	Vaz^263^	NCT03466450
	Vismodegib + IR + TMZ	Newly diagnosed w/nonmethylated MGMT	Ongoing	Wick^239^	NCT03158389
	Vismodegib	Recurrent GBM	Ineffective as monotherapy	Sloan^264^	NCT00980343
VEGF	Bevacuzimab + IR	Recurrent	Improved PFS, no improved OS	Tsien^265^	NCT01730950
Proteosome	Bortezimab + IR + TMZ	Newly diagnosed	Effective for MGMT methylated GBM	Kong^261^	NCT00998010
PARPi	Olaparib + Cediranib maleate	Recurrent	No improved benefit to Bevacuzimab alone	Arrillaga-Romany^266^	NCT02974621
	Olaparib + IR + TMZ	Newly diagnosed	Ongoing	Leseur^254^	NCT03212742
